# Microstructural details of spindle-like lithium titanium phosphate revealed in three dimensions†

**DOI:** 10.1039/d1ra05754e

**Published:** 2021-10-26

**Authors:** Qian Zhang, Roland Schierholz, Krzysztof Dzieciol, Shicheng Yu, Hermann Tempel, Hans Kungl, Rüdiger-A. Eichel

**Affiliations:** Institute of Physical Chemistry, RWTH Aachen University Landoltweg 252074 Aachen Germany; Forschungszentrum Jülich GmbH, Institute for Energy and Climate Research: Fundamental Electrochemistry (IEK-9) Wilhelm-Johnen-Straße D-52425 Jülich Germany r.schierholz@fz-juelich.de

## Abstract

Lithium titanium phosphate LiTi_2_(PO_4_)_3_ is an electrode material for lithium-ion batteries with a specific capacity of 138 mA h g^−1^. Owing to its potential of 2.5 V *vs.* Li/Li^+^ it provides an electrochemically stable interface when used as an anode in all-solid state batteries with NASICON type lithium aluminium titanium phosphate electrolyte. High performance has been identified for *in situ* carbon coated LiTi_2_(PO_4_)_3_ synthesized *via* a hydrothermal route, resulting in micro-scaled spindle shaped particles consisting of nano-scaled sub-particles. To elucidate the internal microstructure of these spindle-like particles in three dimensions we applied tomographic Focused Ion Beam – Scanning Electron Microscopy. For more detailed chemical analysis we performed electron-energy loss spectroscopy and energy dispersive X-ray spectroscopy in the scanning electron microscope as well as high resolution (scanning) transmission electron microscopy for structural insight. It could be clearly shown that the spindle-like particles mainly are made up of LiTi_2_(PO_4_)_3_ sub-particles in the 100 to 400 nm range. Additionally, two types of secondary phase materials were identified. LiTiOPO_4_, which shows different surface morphology, as a volume component of the spindles and TiO_2_ nanoparticles (anatase), which are not only present at the particle surface but also inside the spindle, were detected. Reconstruction from tomography reveals the nanoparticles form a three-dimensionally interconnected network even though their phase fraction is low.

## Abstract 2

The microstructure of spindle-like LiTi_2_(PO_4_)_3_ particles was investigated on the nanoscale in three dimensions by a combination of FIB-SEM tomography with (S)TEM-EDS and -EELS.

## Graphical abstract

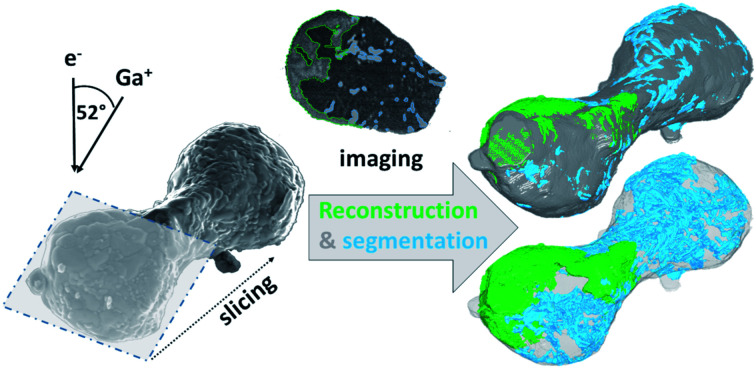

## Introduction

Lithium-ion batteries are the most common energy storage system for electro mobility and portable electronic devices. Among all the novel battery systems, all-solid-state lithium-ion batteries are considered as ideal energy storage devices, which not only improve safety but also require less capsuling and notionally allowing the reduction of overall battery weight.^1–5^

However, also for solid state electrolytes the choice of electrode materials is limited by the electrochemical stability window,^6^ which is kinetically extended as decomposition products form a solid electrolyte interface.^7^ NASICON-type Li_1+*x*_Al_*x*_Ti_2-*x*_(PO_4_)_3_ (LATP) provides high Li-ion conductivity up to 10^−3^ S cm^−1^ at room temperature^8,9^ with stability under air^10^ and an electrochemical stability window from 2.17 V to 4.21 V.^7^ An anode material suiting this stability window and providing the same structure is pure LiTi_2_(PO_4_)_3_ (LTP), without the substitution of Ti^4+^ by Al^3+^.^11,12^ The potential of the (de-) intercalation of Li in LiTi_2_(PO_4_)_3_ (eqn (1)) is 2.32 V, and involves two Li^+^ and two electrons.

1 Li_1_Ti_2_(PO_4_)_3_ + 2e^−^ + 2Li^+^ ↔ Li_3_Ti_2_(PO_4_)_3_

With lithium vanadium phosphate as cathode material all-solid-state aluminium phosphate batteries were demonstrated.^13–15^

For an application as anode material the electrical conductivity can be increased by carbon coating, which can be achieved *in situ* for LiTi_2_(PO_4_)_3_ prepared by solvothermal synthesis.^16^ In addition to carbon coating, which increases the electronic conductivity, the solvothermal synthesis allows to nanostructure the morphology in order to compensate the lower ionic conductivity compared to its Al-substituted form (LATP) used as electrolyte and enhance the performance. The electrochemical performance of spindle-like LiTi_2_(PO_4_)_3_ particles, synthesized using the solvothermal route, showed enhanced cycling stability in a liquid electrolyte cell compared to sol–gel synthesized material without special microstructure.^17^ It was reported that the hydrothermal method paved the way to synthesize lithium titanium phosphate particles with controllable spindle-like shape LiTi_2_(PO_4_)_3_ particles and a formation mechanism was proposed based on scanning electron microscopy (SEM) images recorded after interrupting the solvothermal process.^17^ By X-ray diffraction (XRD) the rhombohedral structure of LiTi_2_(PO_4_)_3_ (ICSD-95979) was confirmed.^18^ For calcination temperatures between 800 °C and 900 °C no secondary phases were reported.^17^ Depending on calcination temperature in literature TiO_2_, Ti_7_O_13_, TiPO_4_, TiP_2_O_7_ and Li_3_PO_4_ and TiP_2_O_7_ were reported to occur as secondary phases with LiTi_2_(PO_4_)_3_.^19–21^ In addition, nanoparticles on the surface of the spindle-like particles were observed by photogrammetry.^22^

In this work we investigate the complete 3-dimensional microstructure of the spindle-like particle agglomerate with focused ion beam (FIB) – SEM tomography. The three different phases were identified by high-resolution (scanning) transmission electron microscopy (HR(S)TEM) and energy dispersive X-ray spectroscopy (EDS) and electron energy-loss spectroscopy (EELS).

## Experimental

### Material synthesis

In this work we use the same material, which was used in previous report.^17^ The spindle-like LiTi_2_(PO_4_)_3_ particles are synthesized by solvothermal method. The precursor solution was prepared by drop-wise addition of titanium isopropoxide, ammonium hydroxide and surface agent. The solvothermal synthesis was performed in a high-pressure reactor at different temperature conditions. The yield of spindle like particles was in the range from 83% to 92%. The final LiTi_2_(PO_4_)_3_ particles are obtained after annealing at 800 °C under argon atmosphere.^17^ The XRD-pattern of the material investigated in this study is shown in Fig. S1† and it appears phase pure.

### Scanning electron microscopy and focused ion beam

For SEM and FIB a small amount of powder was placed on a Si-wafer and a drop of ethanol was applied to disperse it. SEM as well as FIB-SEM tomography were performed on a Helios Nanolab 460 F1, FEI, Netherlands.^23^ SEM images were recorded using Everhart–Thornley detector (ETD) for secondary electrons (SE) giving topographic information and the circular back-scatter (CBS) detector for backscattered electrons (BSE) yielding to Z-contrast. For the slice & view experiment, an isolated particle with spindle-like shape and size below 5 μm was selected. The spindle was sliced with gallium ions (30 kV, 9 nA, 4 nm slice thickness) at stage tilt 0° resulting in slices inclined about 38° to the long axis of the spindle. BSE images were recorded also at tilt 0° with the “In Column Detector” (ICD) at 2 kV and 0.8 nA with image resolution of 2048 px times 2048 px and a horizontal field width of 6.35 μm.

### (Scanning) transmission electron microscopy

TEM images and diffraction patterns were recorded on a Tecnai, FEI, Netherlands at 200 kV.^24^ (S)TEM in combination with EELS and EDS experiments were conducted on a Titan Creweley 80–200, FEI, USA at 80 kV and 200 kV with windowless super-X detectors and a Gatan Enfinium 977 ER spectrometer with 2.5 mm and 5 mm entrance apertures, electrostatic shutter and advanced dual-EELS spectroscopy modes.^25^

## Results

### Scanning electron microscopy

**Fig. 1:**
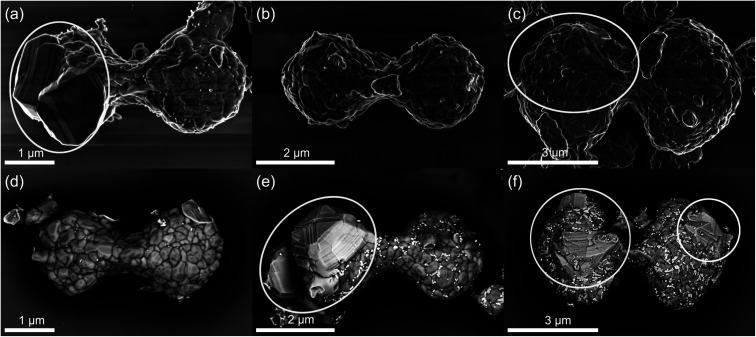
SEM micrographs of spindle like particles of different sizes: small ((a) and (e)), medium ((b) and (d)) and large ((c) and (f)). Regions with different morphology indicating the presence of a secondary phase are marked in (a), (c), (e) and (f).

The morphology of as-synthesized lithium titanium phosphate particles by solvothermal method shown in Fig. 1 is spindle-like, with a variety of sizes: small (a) and (d), medium (b) and (e) and large (c) and (f). The length of most particles is within the range of 4–9 μm with a width between 1 and 4 μm. The surface shows that they are formed by sub-particles of irregular shapes and rounded edges with sizes ranging from 100 nm to 400 nm. The boundaries between the sub-particles appear as dark lines in both SE ((a)–(c)) and BSE ((d)–(f)) micrographs. On all spindle-like particles small nanoparticles are visible at the surface in the size about 50 nm. These nanoparticles appear bright in the BSE micrographs (e) and (f), indicating higher density so they might be presumably attributed to a small amount of TiO_2_ secondary phase reported in literature.^17^

Especially in Fig. 1(e), they seem to be preferentially located at the sub-particle boundaries. Some spindle-like particles show regions with different morphology marked by the white ellipses in Fig. 1(a), (c), (e) and (f). The morphology with well-defined facets and sharp edges differs clearly from the softly shaped surface of the LiTi_2_(PO_4_)_3_ sub-particles, which are also smaller in size. This is evidence for the existence of another, secondary phase with different crystallographic structure. It can be observed in particles of all sizes (Fig. 1(a), (c), (e) and (f)). In BSE-micrographs this secondary phase appears slightly brighter than LTP but darker than the nanoparticles.

### Transmission electron microscopy

SEM provides topographic information (SE) as well Z-contrast (BSE). In the SEM, EDS-analysis is limited as the excitation volume exceeds the size of the microstructural features. (S)TEM provides higher lateral resolution, with probe correction reaching atomic scale. The high-angle annular dark-field (HAADF)-signal is approx. scaling with Z^2^ depending on the collection angle.^26^ In combination with spectroscopy (EDS and EELS) the chemical composition can be analysed on the local scale. HR(S)TEM and electron diffraction give information about the crystalline structure. For this TEM-lamellas of various particles with the thinnest regions <100 nm were prepared by FIB.

### Scanning transmission electron microscopy: energy dispersive X-ray spectroscopy

**Fig. 2:**
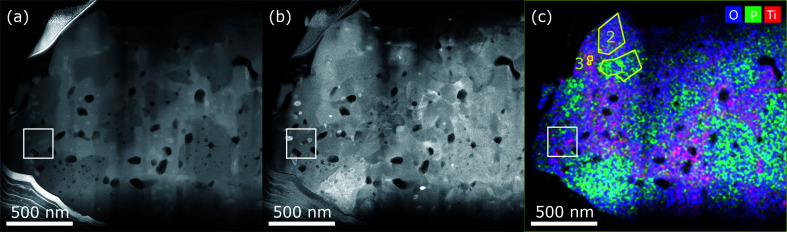
STEM-images of a lamella containing secondary phase: (a) HAADF signal giving Z-contrast (b) DF signal containing diffraction contrast and (c) EDS map showing the net intensity of O (blue), P (green) and Ti (red).

Fig. S2† shows a particle clearly containing the secondary phase with different morphology from which a TEM-lamella was prepared. The volume of the lift out is marked by the grey box. STEM images of the left part of this lamella are shown in Fig. 2(a)–(c). In the HAADF image (a) the secondary phase with different surface morphology can be recognized by its brighter contrast. It is not present only at the surface, but also inside the particle with extended interface with LiTi_2_(PO_4_)_3_. The nanoparticles appear even brighter in the HAADF-signal and are present in both LiTi_2_(PO_4_)_3_ and secondary phase regions. Pores appear dark in the HAADF signal and can be found within both phases and at their boundaries.

The annular dark field (ADF) image in (b) containing diffraction contrast shows the LiTi_2_(PO_4_)_3_ sub-grains. In addition, it shows that also the secondary phase region consists of several sub-particles. Fig. 2(c) displays the superposition of O (blue), P (green) and Ti (red) STEM-EDS net intensities. The P-content is highest in the LiTi_2_(PO_4_)_3_ material. The secondary phase shows less P but higher Ti signal. The nanoparticles do not contain phosphorus and appear purple. From three areas marked yellow, EDS-spectra were quantified. The results are listed in Table S1.† For area 1 this matches well with the theoretical values for LiTi_2_(PO_4_)_3_. In area 2 less P and more Ti is detected. The nanoparticle basically consists only of Ti and O with a ratio of about 0.34, so they are most likely TiO_2_. The white box defines another region of interest (ROI) containing all three phases, from which a more detailed analysis with EDS and EELS was performed at higher magnifications discussed in the following sub section.

### Combined electron energy loss and energy dispersive spectroscopy

**Fig. 3:**
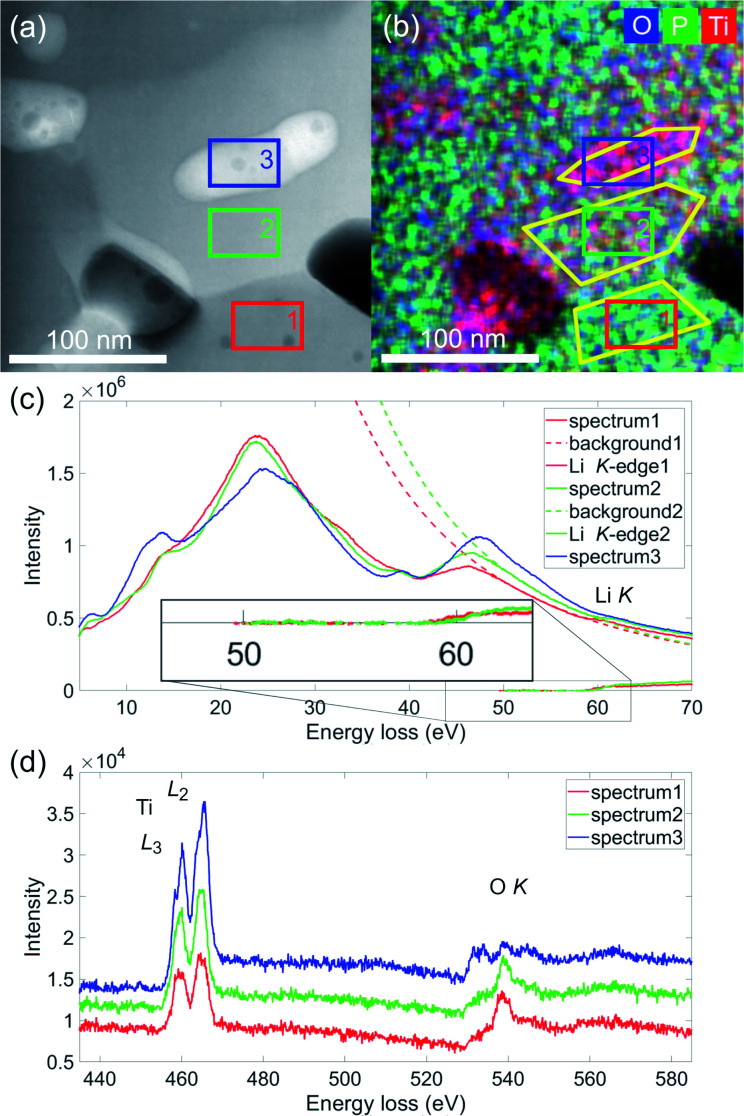
(a) STEM-HAADF signal of the ROI marked in Fig. 2 containing all three phases. The coloured rectangles mark the pixels selected in the spectrum image from which the low loss spectra shown to the right were extracted. (b) Overlay of EDS net intensities of O, P and Ti. Again the coloured rectangles mark the pixels selected in the spectrum image from which the low (c) and core (d) loss spectra shown below were extracted. The yellow framed regions indicate the areas used for EDS-quantification.

Fig. 3 shows the detailed analysis of the ROI containing all three phases framed in Fig. 2 by HAADF, EELS and EDS. The HAADF-signal in (a) indicates and the EDS-net intensities in (b) O (blue), P (green) and Ti (red) prove the presence of three different phases. In Fig. 3(c), the low loss spectra extracted from three different regions of the spectrum image, indicated by the coloured rectangles, are shown. The main feature of the low loss spectra is the plasmon, consisting of several sub peaks. For spectrum 1 (LTP), the plasmon consists mainly of two peaks, the main at ≈24 eV and the second at ≈46 eV. For the secondary phase (spectrum 2), the plasmon appears similar, with a more pronounced shoulder at ≈14 eV and also a slightly more intense second peak. In spectrum 3, obtained from the nanoparticle, the fingerprint of the low loss spectrum looks similar to that of TiO_2_ from the EELS-Atlas as shown in Fig. S3(a),† with clear additional features.^27^

For extraction of the Li K-edge at 55 eV, the background of the decaying Plasmon was fitted with a power law (dashed lines). As the Li K-edge onset appears delayed at 58 eV, an energy window ranging from 50 to 57 eV was used for the fit. This procedure is suitable at least for spectrum 1 and 2. A delayed edge onset is observed in some Li-compounds.^28^ For spectrum 3 the background fit is not applicable, as indicated in Fig. S3(a).† The inset in Fig. 3(c) shows the enlarged extracted signal for spectrum 1 and 2. The signal starting at 58 eV is slightly stronger in spectrum 2, so a possible phase should have higher lithium content than LiTi_2_(PO_4_)_3_.

In Fig. 3(b), the overlay of EDS O, P and Ti background corrected intensities are shown, exposing the different composition of the phases. The quantification of the three yellow marked regions is listed in Table S2.† The results are similar as obtained before in a larger area before in Fig. 2 and Table S1.† For area 1 the quantification matches within experimental error with the theoretical composition of LTP. In area 2, the secondary phase, more Ti than P is present. In the nanoparticle, area 3, mainly Ti and O are present, the amount of P is negligible. Deviations from theoretical composition (TiO_2_) could be explained as the nanoparticle may not fill the complete thickness of the lamella, which was estimated from EELS to be around 55 nm.^29^

In Fig. 3(d), the core loss spectra in the range 435 to 585 eV containing the Ti L-edge (456 eV) and O K-edge (532 eV) are shown. The titanium L-edge neither shifts nor the *L*_3_/*L*_2_-ratio changes, so in all three regions Titanium is present in the 4+ state. But the intensity of the Ti white lines increases from spectrum 1 to spectrum 3. The O K-edge (523 eV) looks similar in spectrum 1 and 2, with a pronounced peak at 540 eV. Such an edge structure was published by Hofer and Golob 1987 for Ca_3_(PO_4_)_2_, which has a similar phosphate structure. A clear difference can be observed in spectrum 3, from the nanoparticle, with three peaks of similar height. This is similar to the fingerprint of the O K-edge in TiO_2_ depicted in Fig. S3(b).†^30^ From the core loss spectra the elemental ratio of Ti and O can be determined and results are listed in Table S2.† For spectrum 1 and 3 the ratios 0.15 and 0.49 match well with LTP (0.167) and TiO_2_ (0.5). For spectrum 2 it is about 0.25 comparable to the EDS-quantification and definitely higher than in LiTi_2_(PO_4_)_3_.

From EDS and EELS we expect the nanoparticles to be TiO_2_ and the unknown secondary phase to be a lithium titanium phosphate containing less phosphorous and more lithium than LiTi_2_(PO_4_)_3._ Looking at the phase diagram (Fig. S4†) calculated with the software materials project^31^ published by Hupfer *et al.*,^32^ the third phase should be either LiTiOPO_4_ or Ti_5_(PO_4_)_3_, as these are the only two phases coexisting with both, LiTi_2_(PO_4_)_3_ and TiO_2_. With the results obtained by EELS, assuming a higher amount of lithium is present in the secondary phase, LiTiOPO_4_ is the only candidate.

### High resolution transmission electron microscopy

**Fig. 4:**
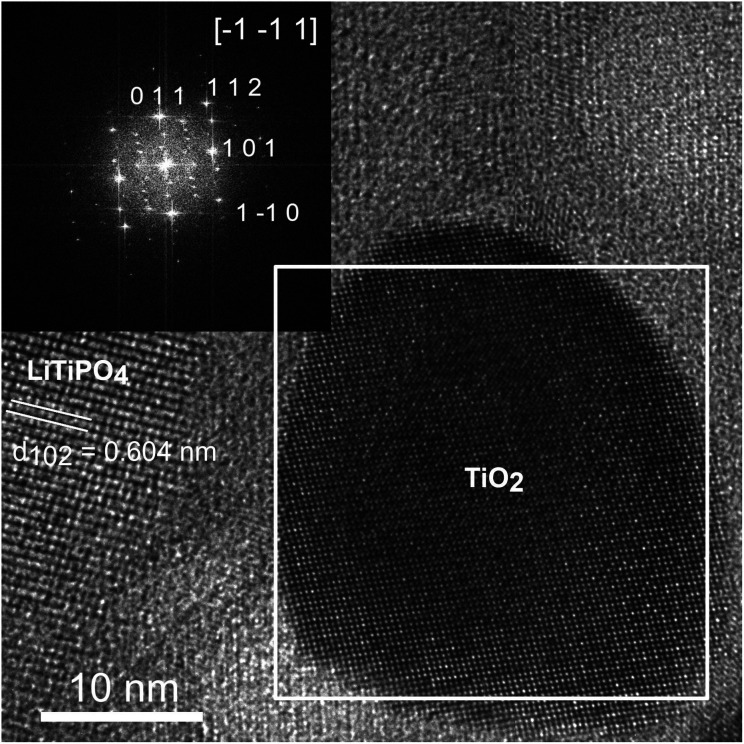
HRTEM image of a nanoparticle in zone axis orientation. The FFT generated from the white box is shown at the inset and can be indexed as [−1 −1 1] zone axis of anatase phase of TiO_2_ (ICSD #9852). In the neighbouring grain lattice plane fringes with a distance of 6.04 Å are observed, which match well with {1 0 2}-planes of the *R*3*c* structure of LTP (ICSD #95979).

Fig. 4 shows a HRTEM image of a TiO_2_ nanoparticle in the LiTi_2_(PO_4_)_3_-matrix. The nanoparticle is close to zone axis orientation and the FFT from the region within the white square is shown on the inset in the upper right. This diffractogram can be indexed as anatase (ICSD #9852)^33^ with zone axis [−1 −1 1]. The lattice spacing observed in left grain of the matrix with *d* = 0.604 nm matches well with *d*_102_ = 0.602 nm of the hexagonal (*R*3̄*c*) structure of LiTi_2_(PO_4_)_3_ (ICSD95979).^18^ It was confirmed by electron diffraction in other areas.

### High resolution scanning transmission electron microscopy

**Fig. 5:**
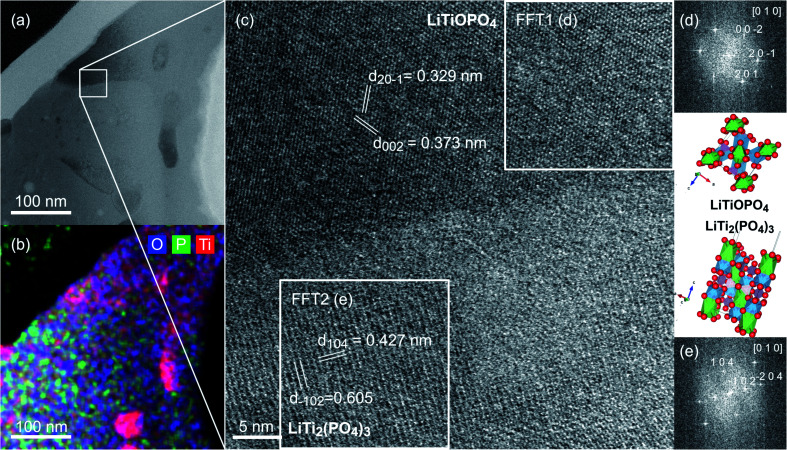
(a) STEM-HAADF image and (b) STEM-EDS map of a boundary between LiTiOPO_4_ and LiTi_2_(PO_4_)_3_. The box in (a) marks the area from which the HRSTEM image in (c) was recorded with both phases being in zone axis orientation as indexed in the FFTs shown in (d) (LiTiOPO_4_) and (e) (LiTI_2_(PO4)_3_) generated from the areas marked by the white squares.

Fig. 5(a) shows a BF-STEM image of a part of another FIB lamella including the secondary phase with different morphology. The boundary between LiTi_2_(PO_4_)_3_ and LiTiOPO_4_ is clearly visible in the BF-signal. The secondary phase region can be recognized by its darker contrast in the BF-image. The EDS-map in Fig. 5(b) shows the different compositions. Also present are TiO_2_ nanoparticles, which were already identified by both EELS & HRTEM. The white box in Fig. 5(a) marks the position from which a HRSTEM image of the boundary between LiTi_2_(PO_4_)_3_ and the secondary phase shown in Fig. 5(c) was recorded. Both grains, LiTi_2_(PO_4_)_3_ in the lower part and the secondary phase in the upper part, are in zone axis orientation as shown by the FFTs' in (d) and (e) of the two marked square regions. From chemical analysis, LiTiOPO_4_ is the possible phase and for this composition several structure models were found in the ICS-database. The best match of FFT1 in Fig. 5(d) with simulated Electron diffraction patterns (CrystBox)^34^ was observed for ICSD #153522.^35^ FFT2 in Fig. 5(e) can be indexed as [0 1 0] with the crystal structure of LiTi_2_(PO_4_)_3_ published by Aatiq *et al.* (ICSD #95979).^18,36^ Next to the two FFT's the crystallographic structures of LiTiOPO_4_ (d) and LiTi_2_(PO_4_)_3_ (e) are shown in the corresponding orientation. The interface between the two phases shows a narrow amorphous region. During HRSTEM imaging an ongoing amorphization was observed, which can be attributed to beam damage. So we cannot draw a conclusion about the atomistic structure of the interface in detail. It might have been completely crystalline, before being irradiated by electrons.

### FIB-SEM tomography

From SEM, only the outer surface of the spindle-like particles is accessible. With FIB-SEM tomography the complete internal microstructure of the particle can be analysed by successive slicing with an ion beam and recording SEM micrographs of each slice. For materials contrast, backscattered electrons were detected with an in-column detector. With the analysis described above, the contrast can be attributed to different phases: pores (black), LiTi_2_(PO_4_)_3_ (dark grey), LiTiOPO_4_ (intermediate grey) and TiO_2_ (bright).

For all 669 slices the histograms were equalized and lateral bias was removed as exemplarily shown for one slice in Fig. S5.† Therefore, multimodal registration was not required for alignment. Instead, grey-level sensitive metric was used together with linear registration scheme. Later, thresholding was applied to delineate the particle.

**Fig. 6:**
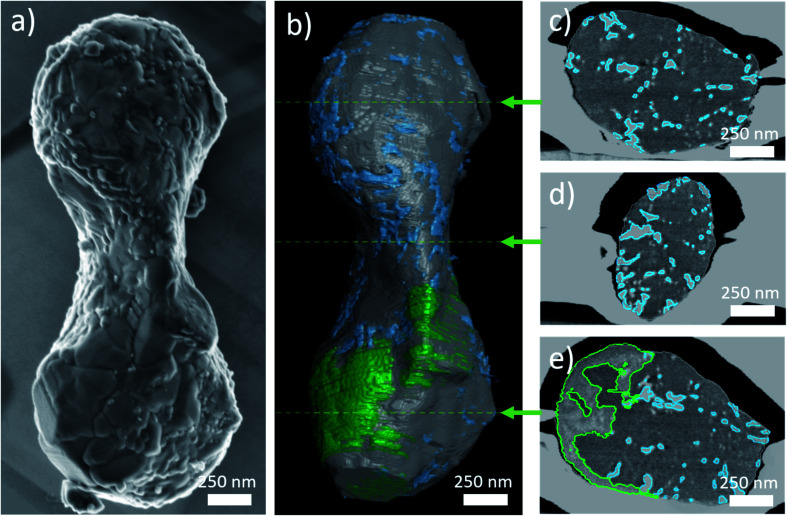
(a) Top view SE image of the particle selected for FIB tomography. (b) Surface rendering of the segmented FIB tomography data. Grey, green and blue colours represent LTP, LiTiOPO_4_ and TiO_2_ respectively. The red arrows indicate the positions of the slices shown in (c)–(e) visualizing the segmentation inside the volume.

Fig. 6 shows an SEM image of one particle selected for FIB-SEM tomography (a) in top view, and segmented FIB reconstruction (b). The main part is dark grey and corresponds to LiTi_2_(PO_4_)_3_. Green color is attributed to LiTiOPO_4_ and the TiO_2_ nanoparticles are rendered blue. Fig. 6(c)–(e) show three exemplary cuts through the volume. To visualize the segmentation process, the outlines for LiTiOPO_4_ and TiO_2_ are overlaid to these slices. As a fourth microstructural feature, pores are present, sparsely distributed within the particle.

Thanks to three-dimensional delineation, volume analysis was possible. It revealed that LiTi_2_(PO_4_)_3_ makes up 86% of the whole spindle volume, LiTiOPO_4_ about 7% and TiO_2_ about 6%. Porosity is below 1%. Moreover, porosity will vary between spindles. In the (S)TEM porosity appears higher, which can be due to sample preparation, *i.e.* the thinning process could increase the number of voids and enlarge the existing ones.

**Fig. 7:**
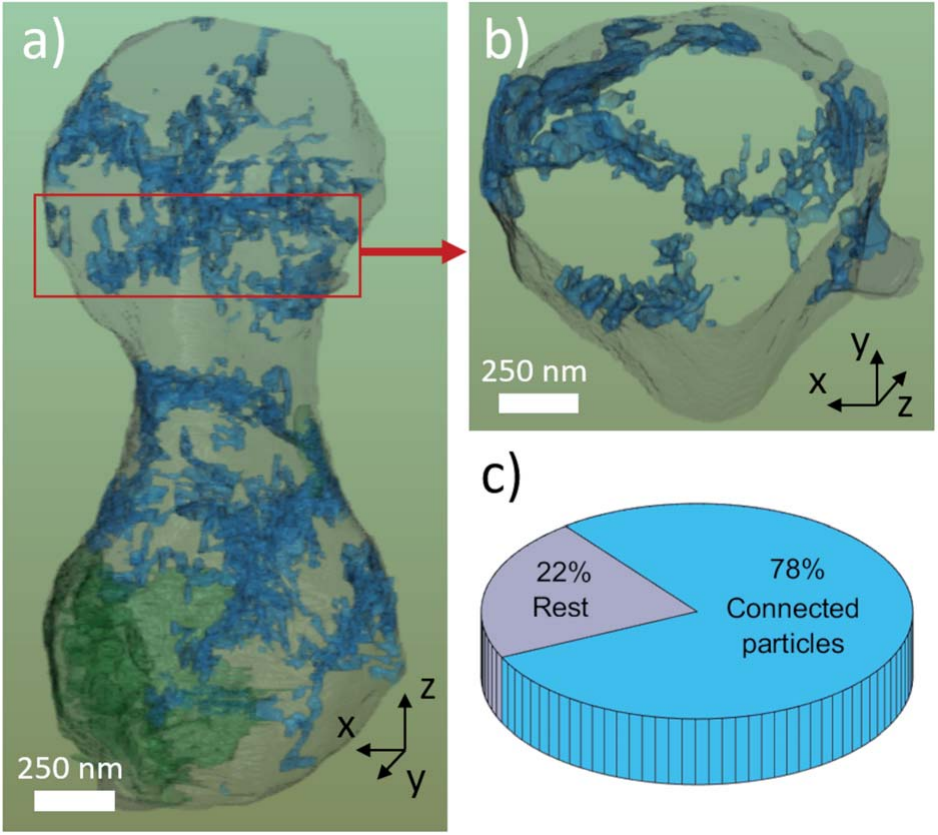
(a) Surface rendering of the three-dimensional network of TiO_2_ nanoparticles (blue) with bulk material (semi-transparent grey) and LiTiOPO_4_ secondary phase (green) displayed for orientation. A longitudinal clipping plane was applied and non-interconnected TiO_2_-nanoparticles were removed for clarity. The single slab of approx. 250 nm thickness (b) as indicated by the red frame in (a) reveals the three-dimensional nature of particles network. The pie chart (c) visualizes the amount of interconnected TiO_2_ particles.

Fig. 7 shows the surface rendering of the TiO_2_ nanoparticles (blue) with the secondary phase LiTiOPO_4_ (green, semi-transparent) and transparent outline of the main phase for the orientation. SEM images of the spindles surface and STEM-ADF revealed already that LiTi_2_(PO_4_)_3_ spindle is formed by sub-particles ≤500 nm, these however cannot be separated by the BSE-contrast used for segmentation. Same applies to the sub-particles of LiTiOPO_4_ revealed by STEM. Therefore, FIB tomography shows only two regions, in which LiTiOPO_4_ is present. In SEM (and STEM), the TiO_2_ nanoparticles appeared isolated on the spindle surface, some of them connected and in general preferentially located at the LTP sub-grain boundaries.

Only by FIB-SEM tomography it can be shown that the majority (78%) of TiO_2_ particles are connected, resulting in one, large particle network. This means that even though the TiO_2_ particles are only a minor phase, they could play an important role for the performance of the LTP spindle as described by Yu *et al.*^17^ The increased cycling capability and higher capacity at high cycling rates, compared to sol–gel synthesized materials, can be achieved by increased Li-diffusion at (i) the LiTi_2_(PO_4_)_3_ sub-grain boundaries or (ii) the TiO_2_ network. Anatase TiO_2_ has been reported as anode material with reversible capacity of 168 mA h g^−1^ and fast kinetics for nanostructured material.^37–39^ The role of the (iii) LiTiOPO_4_ secondary phase is however not clear. To put more delight on this question, further experiments (*e.g. in situ*) are required which go beyond the scope of this paper.

## Conclusions

This work shows the importance of a multimodal, three-dimensional microscopic investigation. Although minor secondary phases can be detected by bulk methods, *e.g.* XRD, no conclusion about the topology of present phases could be drawn. Microscopy can provide this insight and is more sensitive for the detection of minor secondary phases. SEM revealed the presence of LiTi_2_(PO_4_)_3_ sub-particles in the 100 to 400 nm range of undefined shapes, which form the spindle like morphology. Additionally, the presence of two minor phases on the surface, one with a different surface morphology with sharp edges, and the other in form of nanoparticles, were observed. For a detailed chemical and structural investigation high resolution (scanning-) transmission electron microscopy in combination with Electron Energy-Loss Spectroscopy and Energy Dispersive X-ray Spectroscopy was applied. This was necessary to attribute the contrast observed in FIB-SEM tomography to LiTiOPO_4_ and TiO_2_ (anatase). STEM on FIB-cut lamellae gave an impression on the internal distribution, especially of the LiTiOPO_4_ secondary phases. Only FIB-SEM tomography could give a complete 3-dimensional analysis on the nanoscale and reveal that the majority of TiO_2_ nanoparticles form an interconnected network inside the spindle, even though they make only about 5% of the volume.

## Author contributions

QZ: SEM, FIB-SEM, TEM, funding CSC; RS: conceptualization, investigation (STEM-EDS and STEM-EELS), supervision, writing, visualization, validation; KD: tomography reconstruction, visualisation, software, writing; SY: conceptualization and synthesis of the material; HT supervison; HK: supervision, writing; RAE: funding; writing, supervision.

## Conflicts of interest

There are no conflicts to declare.

## Supplementary Material

RA-011-D1RA05754E-s001
