# Supplementary material for: Microstructural details of spindle-like lithium titanium phosphate revealed in three dimensions
Source: RSC Adv. 2021 Oct 26;11(55):34605–12. doi: 10.1039/d1ra05754e (PMC9042717; doi:10.1039/d1ra05754e)
Supplement: RA-011-D1RA05754E-s001 [file RA-011-D1RA05754E-s001.pdf]

Electronic Supplementary Material (ESI) for RSC Advances.
This journal is © The Royal Society of Chemistry 2021

Supporting Information

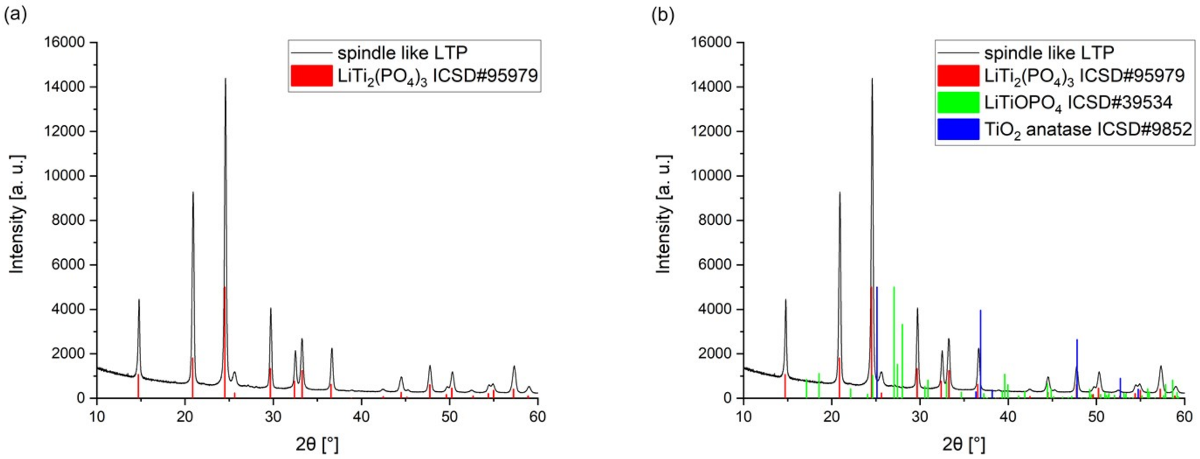

Figure S1: XRD-pattern of spindle like $LiTi_2(PO_4)_3$ [17] calcined at 800 °C.

Figure S1 shows the XRD-pattern of the investigated sample charge. When only a single phase is considered ($LiTi_2(PO_4)_3$ (ICSD#95979), Figure S1 (a)) phase purity can be assumed. Some minor humps are visible around 27° and 28° but these cannot be identified as clear secondary phase reflections for phase matching. With the knowledge based on secondary phase identification by (S)TEM in Figure S1 (b) these weak humps can be attributed to the two strongest reflections of $LiTiOPO_4$ (ICSD#39534, green bars). The three reflections of anatase (ICSD#9852, blue bars) overlap with the reflections of $LiTi_2(PO_4)_3$, thus, small amounts of anatase are difficult to detect as secondary phase in LTP by XRD.

Figure S2 one particle with the 2 µm wide central part marked by a box indicating the lift-out for TEM-lamella preparation. STEM images of this lamella are shown in Figure 2 and 3.

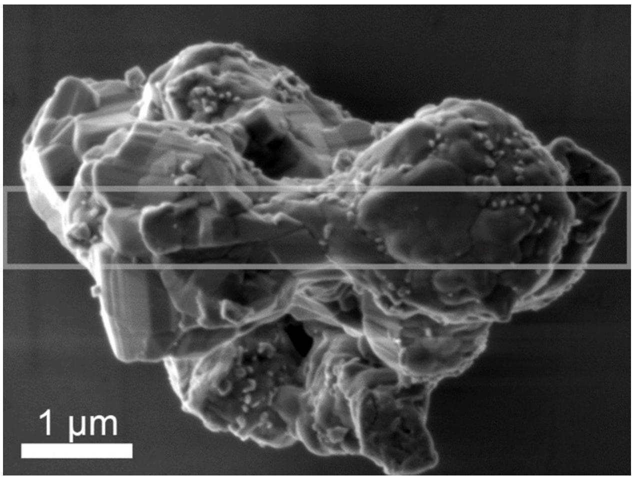

Figure S2: (a) SEM top view image of a spindle-like particle containing the secondary phase with different morphology in the left part, from which a TEM-lamella was lifted out as indicated by the framed box.

Table S1 list the theoretical compositions, excluding lithium, which cannot be detected by EDS, for $LiTi_2(PO_4)_3$, and $LiTiOPO_4$ and TiO_2, which were attributed to the secondary phase and the nanoparticles respectively. Also listed are the results of quantification from the ED-spectra from the three areas marked in Figure 2. Table S2 summarizes the results obtained from Quantification of EDS from the three areas marked in Figure 3(b) and EELS core loss spectra from the three boxes marked in Figure 3(a).

Table S1: EDS quantification of the three areas marked in Figure 2.

	O [at%] (excl. Li)	P [at%] (excl. Li)	Ti [at%] (excl. Li)	Atomic ratio Ti/O
**$LiTi_2(PO_4)_3$**	70.6	17.7	11.8	0.167
**$LiTiOPO_4$**	71.4	14.3	14.3	0.2
**TiO_2**	66.7	0.0	33.3	0.5
**Area 1**	75.5±5.8	14.3±2.9	10.1±1.5	0.134±0.030
**Area 2**	75.8±5.6	10.2±2.1	14±2.1	0.185±0.041
**Area 3**	71.7±7.6	3.7±0.9	24.6±4.1	0.343±0.094

Table S2: EDS- and EELS quantification of the three areas marked in Figure 3b.

	O [at%] (excl. Li)	P [at%] (excl. Li)	Ti [at%] (excl. Li)	Atomic ratio Ti/O
**EDS**				
**Area 1**	71.8±6.1	16.2±3.4	12±2	0.17±0.04
**Area 2**	70.1±6.2	12.1±2.5	17.7±2.8	0.25±0.06
**Area 3**	70.2±6.8	5.7±1.2	24.1±4	0.34±0.09
**EELS**				
**Area 1**	86.95		13.05	0.15±0.021
**Area 2**	79.86		20.14	0.25±0.036
**Area 3**	67.15		32.85	0.49±0.069

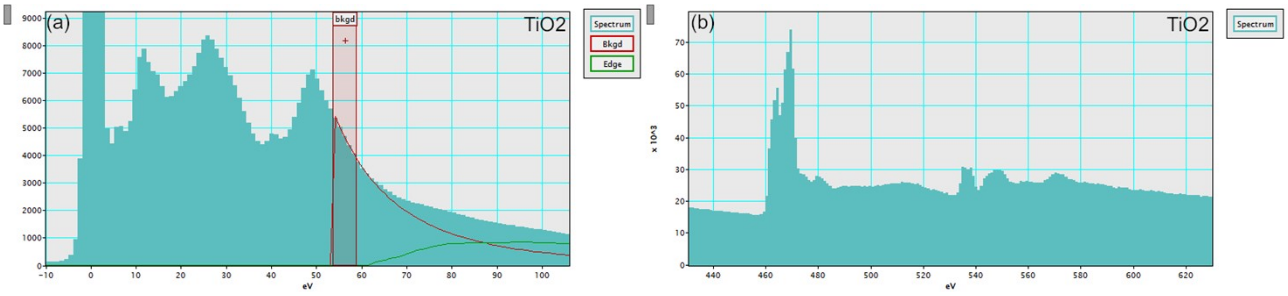

Figure S3: (a) low loss spectrum of TiO_2 from the EELS-atlas. (b) Core loss spectra of TiO2 taken from the EELS atlas.

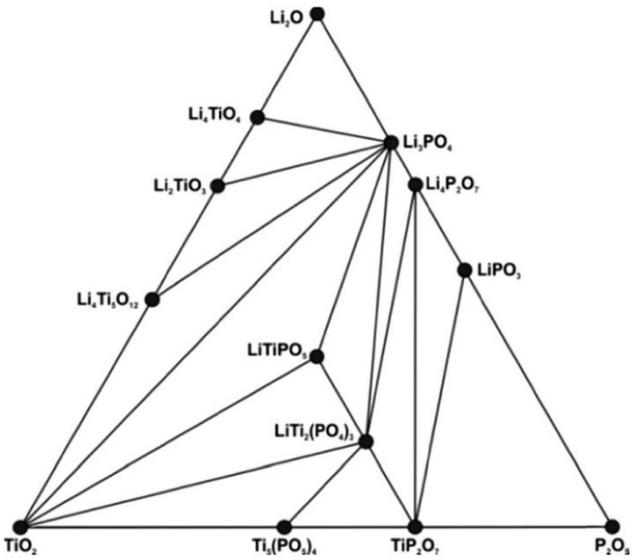

Figure S4: Quasi-ternary phase diagram Li_2O-TiO_2-P_2O_5 calculated with materials project reused from reference 32.

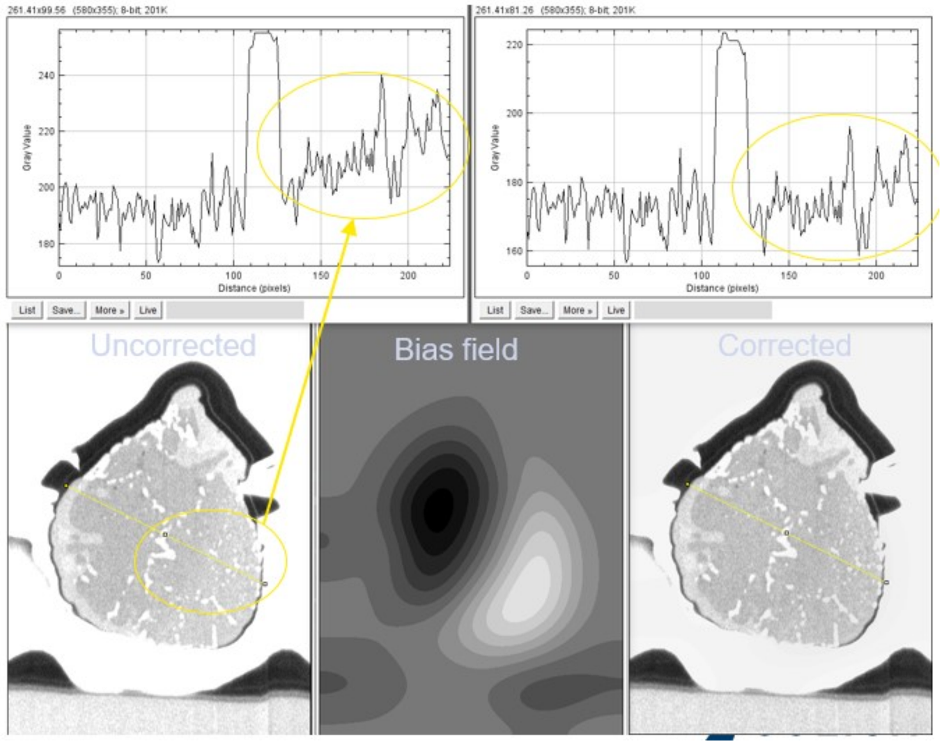

Figure S5: (top) Histograms equalization and (bottom) removal of lateral bias.
